# Genistein Combined Polysaccharide (GCP) Can Inhibit Intracrine Androgen Synthesis in Prostate Cancer Cells

**DOI:** 10.3390/biomedicines8080282

**Published:** 2020-08-11

**Authors:** Neelu Batra, Anhao Sam, Tibebe Woldemariam, George Talbott, Ralph W. de Vere White, Paramita M. Ghosh, Nilesh W. Gaikwad, Simeon O. Kotchoni, Ruth L. Vinall

**Affiliations:** 1 Department of Pharmaceutical & Biomedical Sciences, California Northstate University College of Pharmacy, Elk Grove, CA 95757, USA; nbatra@UCDAVIS.EDU (N.B.); Anhao.Sam6546@cnsu.edu (A.S.); TWoldemariam@cnsu.edu (T.W.); gctalbott@gmail.com (G.T.); simeon.kotchoni@cnsu.edu (S.O.K.); 2 Department of Biochemistry and Molecular Medicine, UC Davis, Sacramento, CA 95817, USA; paghosh@ucdavis.edu; 3 UC Davis Comprehensive Cancer Center, UC Davis, Sacramento, CA 95817, USA; rwdeverewhite@ucdavis.edu; 4 Department of Urological Surgery, UC Davis, Sacramento, CA 95817, USA; 5 VA Northern California Health Care System, Mather, CA 95655, USA; 6 Gaikwad Steroidomics Laboratory, LLC, Davis, CA 95616, USA; nilesh@gaikwadsteroidomics.com

**Keywords:** prostate cancer, Intracrine androgen synthesis, CYP17, genistein combined polysaccharide, Genistein

## Abstract

Our group and others have previously shown that genistein combined polysaccharide (GCP), an aglycone isoflavone-rich extract with high bioavailability and low toxicity, can inhibit prostate cancer (CaP) cell growth and survival as well as androgen receptor (AR) activity. We now elucidate the mechanism by which this may occur using LNCaP and PC-346C CaP cell lines; GCP can inhibit intracrine androgen synthesis in CaP cells. UPLC-MS/MS and qPCR analyses demonstrated that GCP can mediate a ~3-fold decrease in testosterone levels (*p* < 0.001) and cause decreased expression of intracrine androgen synthesis pathway enzymes (~2.5-fold decrease of 3βHSD (*p* < 0.001), 17βHSD (*p* < 0.001), CYP17A (*p* < 0.01), SRB1 (*p* < 0.0001), and StAR (*p* < 0.01)), respectively. Reverse-phase HPLC fractionation and bioassay identified three active GCP fractions. Subsequent NMR and LC-MS analysis of the fraction with the highest level of activity, fraction 40, identified genistein as the primary active component of GCP responsible for its anti-proliferative, pro-apoptotic, and anti-AR activity. GCP, fraction 40, and genistein all mediated at least a ~2-fold change in these biological activities relative to vehicle control (*p* < 0.001). Genistein caused similar decreases in the expression of 17βHSD and CYP17A (2.5-fold (*p* < 0.001) and 1.5-fold decrease (*p* < 0.01), respectively) compared to GCP, however it did not cause altered expression of the other intracrine androgen synthesis pathway enzymes; 3βHSD, SRB1, and StAR. Our combined data indicate that GCP and/or genistein may have clinical utility and that further pre-clinical studies are warranted.

## 1. Introduction

Between 20 and 50% of prostate cancer (CaP) patients will experience disease recurrence within 10 years following first line treatment (typically prostatectomy or radiation therapy) and be placed on leutenizing hormone releasing hormone (LHRH) agonist-based androgen deprivation therapy (ADT) [1,2,3]. ADT inhibits systemic androgen production and thereby slows prostate cancer cell proliferation and disease progression by limiting testosterone (T) and dihydrotestosterone (DHT)-mediated activation of the androgen receptor (AR) [4]. Unfortunately, the majority of patients fail ADT and develop castration-resistant prostate cancer (CRPC) within 2 years [5]. At this point, most patients are treated with cytotoxic chemotherapy and/or AR signaling pathway inhibitors and the median progression-free survival is estimated at only ~14 months [6]. Intracrine androgen synthesis by CaP cells has been shown to contribute to failure of ADT by allowing for autocrine activation of AR. Abiraterone, an inhibitor of the intracrine androgen synthesis enzyme CYP17A, has been approved to treat CPRC patients and was recently approved for combination with ADT in patients with metastatic hormone-sensitive CaP [7,8].

Our group and others have previously demonstrated that genistein combined polysaccharide (GCP), a soy isoflavone-rich contemporary and alternative medicine (CAM), can inhibit CaP cell proliferation and induce apoptosis in cell line and animal models of CaP [9,10,11,12,13,14,15,16,17,18]. GCP is produced by culturing soybean with mycelia from the *Ganoderma lucidum* mushroom which produces beta-glucosidase, an enzyme that can cleave the glucoside forms of isoflavones into more easily bioabsorbed aglycone forms [19]. The primary isoflavones present in GCP are genistein (93 mg/g), daidzein (57 mg/g) and glycetein (~20 mg/g). Several groups, including ours, have demonstrated that GCP can inhibit growth of CaP cells both in vitro and in vivo through inhibition of AR activity [9,10,11,12,13,14,15]. AR is well known to be the main driver of androgen-sensitive CaP cell proliferation [20]. The precise mechanism by which GCP inhibits AR activity has not previously been reported. Possibilities include inhibition of intracrine androgen synthesis by CaP cells, inhibition of ligand binding to AR, inhibition of AR nuclear translocation, and/or inhibition of AR co-activators.

In the current study, we used UPLC-MS/MS and qPCR to determine whether GCP impacts intracrine androgen synthesis. Therapies which inhibit intracrine synthesis have the potential to extend the timeframe between disease recurrence following first line treatment and ADT failure, and thereby improve CaP survival rates. An advantage of using CAMs in this setting is that they have a favorable toxicity profile and are generally well accepted by patients. A disadvantage is that the implementation of clinical studies using CAMs can be challenging because often they are a complex mix and the active component may be unknown. To address this issue, we performed HPLC fractionation and bioassays followed by NMR analysis to allow for identification of the active component of GCP.

## 2. Experimental Procedures

### 2.1. Chemicals

GCP was generously provided by Amino Up Chemical company, Ltd. (Sapporo, Japan) but is commercially available. Genistein, glycitein and daidzein were purchased from Cayman Chemicals (Ann Arbor, MI, USA). All stock solutions of GCP, genistein, glycitein, and daidzein were made in 50% DMSO/50% ethanol. HPLC grade methanol, trifluoroacetic acid and acetonitrile (Sigma-Aldrich, St. Louis, MO, USA). MTT reagent (Thiazolyl Blue Tetrazolium) was purchased from Sigma-Aldrich (St. Louis, MO, USA). DMSO and ethanol were purchased from EMD Millipore (Burlington, MA, USA). All of the mouse monoclonal antibodies and horseradish peroxidase-linked anti-mouse secondary antibodies were purchased from Cell Signaling (Beverly, MA, USA). The GAPDH antibody was purchased from EMD Millipore (Burlington, MA, USA).

### 2.2. GCP Extraction, Fractionation, and UPLC-MS Analysis

Five milliliters of extraction mixture (Methanol:Water, 80:20 *v*/*v*) were added to 100 mg GCP and vortexed for 5 min. The mixture was then centrifuged at 2000 rpm for 10 min, and the supernatant filtered and used for subsequent Reverse-Phase HPLC analysis (HPLC: Alliance 2790 separations module attached with 996 Photodiode Array detector and Fraction collector-III (Waters Corporation, Milford, MA, USA). For the Reverse-Phase HPLC analysis, the analytical column was a YMC-Pak (250 × 6.0 mm i.d.), using methanol water (80:20) as a mobile phase at a flow rate of 1ml/minutes for 60 min. The effluent was monitored using a photodiode array from wavelengths from 210 to 400 nm. Waters fraction collector III (Waters Corporation, Milford, MA, USA) was used to collect fractions every minute. The fractions were dried in Labconco (Kansas City, MO, USA) freeze dryer and suspended in Ethanol:DMSO, 50:50 *v*/*v*. Fifty microliters of the GCP supernatant were loaded on the column.

UPLC-MS analyses of GCP eaxtract and HPLC fractionated samples were conducted using a Waters Acquity UPLC system connected with triple quadruple mass spectrometer (Waters, Milford, MA, USA) using Electro Spray Ionization (ESI) in positive (PI) mode, capillary voltage of 3.0 kV, extractor cone voltage of 3 V, sample cone voltage of 32 V, and detector voltage of 500 V. Desolvation gas flow was maintained at 600 L/h. Source temperature and desolvation temperatures were set at 150 and 350 °C, respectively. Analytical separations on the UPLC system were conducted using an Acquity UPLC C18 1.6 µm column (2 X 150 mm) at a flow rate of 0.15 ml/min. The elutions from the UPLC column were introduced to the mass spectrometer. Resulting data were analyzed and processed using MassLynx 4.1 software.

### 2.3. Cell Culture

LNCaP cells were acquired from American Type Culture Collection (ATCC, Manassas, VA, USA). PC346C was generously provided by Dr. W.M. van Weerden (Josephine Nefkens Institute, Erasmus MC, Rotterdam, Netherlands). Both of these CaP cell lines are androgen-sensitive and express AR [21]. The LNCaP cell line was originally derived from a lymph node metastasis of CaP. LNCaP cells harbor an AR point mutation (T877A) which makes them unresponsive to anti-androgens [22]. The PC-346C cell line was derived from the transurethral resection of a primary prostate tumor [23]. PC-346C cells express wildtype AR. All cell lines were maintained cultured in RPMI 1640 media (Invitrogen, Carlsbad, CA, USA) supplemented with 10% FBS (Omega, Tarzana, CA, USA) and 1% pen/strep (Invitrogen, Carlsbad, CA, USA) in a humidified environment of 5% CO_2_ in air.

### 2.4. Proliferation Assays

For proliferation assays, 10,000 cells were seeded per well in a 24-well tissue culture plate in 500 µL of RPMI media and allowed to attach overnight. Cells were then treated with vehicle, GCP (100 µg/mL), genistein (9 µg/mL), daidzein (6 µg/mL), glycitein (2 µg/mL), or GCP fractions (concentration used reflected their relative concentration in 100 µg/mL GCP) and incubated for 72 h. Cell proliferation was evaluated using the MTT assay as previously described [15]. Three independent experiments were performed.

### 2.5. Immunoblot Analysis

Protein extraction, quantification, and Western blot were performed as described previously [15]. Briefly, cellular protein extracts were resolved on SDS-PAGE and proteins were transferred to nitrocellulose membranes. After blocking for 1 h at room temperature in 5% milk in TBS/0.1% Tween 20 (TBST), membranes were incubated overnight with primary antibodies (androgen receptor, PSA, or GAPDH antibody) at 4 °C. After washing with TBST, membranes were incubated with horsearadish peroxidase-linked anti-mouse secondary antibody, washed again with TBST, and then incubated with enhanced chemiluminescence (ECL) reagent (Amersham Pharmacia, Piscataway, PA, USA). The membranes were visualized using a Chemi doc imaging system (Bio-Rad, Hercules, CA, USA). Three independent experiments were performed.

### 2.6. Apoptosis Analysis

LNCaP cells (5 × 10^5^) were seeded into 10-cm dishes and allowed to adhere overnight. Cells were then treated with GCP, genistein, glycitein, and GCP fractions. The concentration of genistein, glycitein, and GCP fractions was adjusted to reflect their relative concentration in GCP (100 µg/mL). At 72 h posttreatment, cells were harvested by trypsinization for cell cycle analysis. Levels of apoptosis were measured by staining cells with propidium iodide and then assessing fractional DNA content using flow cytometry as described previously [15]. The percentage of cells in the sub-G_1_ phase, i.e., the percentage of cells with fractional DNA content, was determined from the DNA histogram obtained from each cell sample using Phoenix Multicycle software (Phoenix Flow Systems, San Diego, CA, USA). Three independent experiments were performed.

### 2.7. Reverse Transcription Quantitative Real-Time PCR

Total RNA was extracted using Direct-zol RNA isolation kit (Zymo Research, Irvine, CA, USA). A Nanodrop (ThermoFisher Scientific, Waltham, MA, USA) was used to quantify RNA; 500 ng of total RNA was used for cDNA synthesis. The NxGen M-MuLV reverse transcriptase kit (Lucigen, Middleton, WI, USA) was used to generate cDNA. The BioRad SYBR Green kit and specific primers for molecules required for intracrine androgen synthesis (3βHSD, 17βHSD, CYP17A, SRB1, StAR) [24] were used for qPCR reactions. QPCR analysis was carried out on a CFX96 Touch real-time PCR system (Bio-Rad, Hercules, CA, USA). Levels of mRNA were normalized to βactin and were analyzed using the 2^−ΔΔCT^ method. Reactions were run in triplicate in three independent experiments.

### 2.8. Sample Preparation and UPLC-MS/MS Analysis of Steroid Metabolites

LNCaP cells were treated with vehicle and GCP (100 µg/mL) for 72 h. Cells were cultured in serum-free and phenol-free media for 24 h prior to collection of media for mass spectrometry analysis. Thirty million cells were collected from vehicle versus GCP treated cells and stored at −80 °C until further use. The steroid extraction and analysis were performed as described previously [25,26] using triple quadruple mass spectrometer (Waters Corporation, Milford, MA, USA) connected with Waters Acquity UPLC system. Analytical separations were performed using an ACQUITY UPLC C18 (1.6 μm 1 × 150 mm) analytical column. Resulting data was processed by using TargetLynx 4.1 software (Waters Corporation, Milford, MA, USA).

### 2.9. NMR Analysis of Fraction 40

1H- and HSQC spectra were measured on a Brucker 600 AV III with 5 mm TCI probe (Temp, 303.7-6) spectrometer (Brucker, Billerica, MA, USA) using DMSO as a solvent and TMS as an internal standard and chemical shifts are given in δ (ppm).

### 2.10. LC-MS Analysis of Fraction 40

In order to validate the major bioactive compound of Fraction 40, we additionally ran this fraction and advanced high-resolution ThermoFisher UltiMate 3000^TM^ UHPLC system RSLC (Dionex, Sunnyvale, CA, USA) coupled to a Bruker Impact HD II QTOF-MS spectrometry operated by Hystar software and empowered with integrated state-of-the art data analysis software pipeline (Brucker, Billerica, MA, USA). For adequate performance of the instrument and a quality control (QC) of the LC-MS run method, a blank (made of 75% methanol (*v*/*v*) and 0.1% formic acid (*v*/*v*)) was always included in the LC-QTOF MS run at the beginning of each run. For reproducibility, six replicate runs were done per sample. A total of 10 μL of fraction 40, the authentic compound (as positive control) and the blanc were injected per run, respectively. The data acquisition was performed using a reverse phase (RP) Zorbax Eclipse Plus 3.5 μm poroshell C-18 column (3.5 μm, 4.6 × 100 mm). The sample was separated using a 20 min gradient LC method with acetonitrile (ACN) and water (H_2_O) mobile phases (both containing 0.1% formic acid) at a flow rate of 0.4 mL/min. The gradient started at 1 min from ACN/H2O (2%/98%), followed by a linear gradient to reach ACN/H2O (98%/2%) in 10 min, and held for 2 min at ACN/H_2_O (98%/2%) followed by 1 min to reach ACN/H2O (2%/98%) and then kept for 6 min for re-equilibration at ACN/H2O (2%/98%).

For QTOF MS data acquisition, full scan mass spectra (*m/z* 20–1000) were measured in positive electrospray ionization (ESI) mode, and the mass spectrometer was operated with the following parameters: capillary, 4.5 kV; nebulizer pressure, 2.0 bar; dry gas flow, 8.0 L/min; dry gas temperature, 200 °C; scan rate, 8 Hz. Sodium (Na) Formate (10 mM) was introduced through a divert valve at the beginning of each chromatographic run for automatic internal calibration. MS/MS data acquisition was acquired using the following additional parameters: number of precursors, 3; absolute threshold, 1500.

For metabolomics data processing, an integrated Bruker DataAnalysis^TM^ 4.3 software was used for extraction ion chromatogram (EIC) of the targeted compound using its known molecular formular or molecular mass. The software will automatically use the embedded Find Molecular Features (FMF) algorithm that combines all molecular features belonging to the same compound (isotopes, charge states, adducts and common neutral losses) if the targeted compound is in the base peak chromatogram (BPC) to reveal the EIC, the MS and MS/MS profile of the target compound. The compound identification (molecular formula determination) was carried out by combined evaluation of mass accuracy, isotopic patterns, adduct and fragment information using a manual SmartFormula software feature. The open source Compound Crowler is then used for an automatic identification of the target compound(s) by means of database online search.

### 2.11. Statistical Analyses

Statistical analyses were performed using GraphPad Prism software. When appropriate, Student’s test and one-way ANOVA with Tukey’s post-hoc analysis were conducted to determine whether differences mediated by each treatment group were statistically significant when compared with other treatment groups and the vehicle control. Statistical significance is defined as *p* < 0.05 or lower.

## 3. Results

### 3.1. GCP Comprises High Levels of Genistein, Daidzein, and Glycitein as well as Other Unknown Compounds

**Figure 1:**
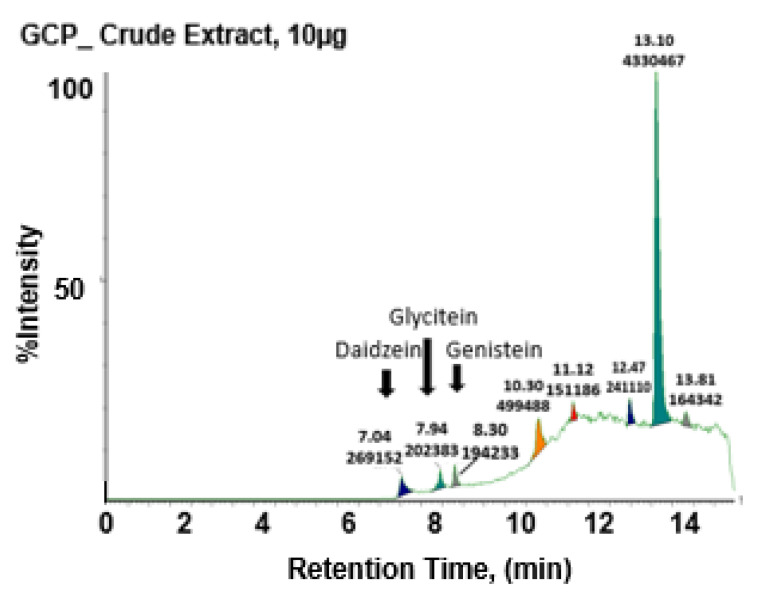
UPLC-MS demonstrate s genistein combined polysaccharide (GCP) comprises of high levels of genistein, daidzein, and glycitein as well as other unknown compounds. Our analysis comparing GCP to genistein, daidzein, and glycitein standards revealed that GCP comprises 9% genistein, 6% daidzein, and 2% glycitein. Multiple other peaks of unknown identity were also observed, demonstrating GCP is a complex mix of compounds.

UPLC-MS analysis of GCP shows that GCP contains high levels of three major isoflavones; genistein, daidzein, and glycitein (9%, 6%, and 2%, respectively; this is consistent with prior reports [19]) (Figure 1). Multiple other peaks of unknown identity were also observed demonstrating that GCP is a complex mix of compounds. Note that for all bioassay experiments the concentrations of the GCP fractions and of genistein, daidzein, and glycitein standards were adjusted to reflect their relative concentration in GCP (100 µg/mL), so that a fair comparison of their activity relative to the activity of GCP was possible.

### 3.2. GCP Inhibits Intracrine Androgen Synthesis

**Figure 2:**
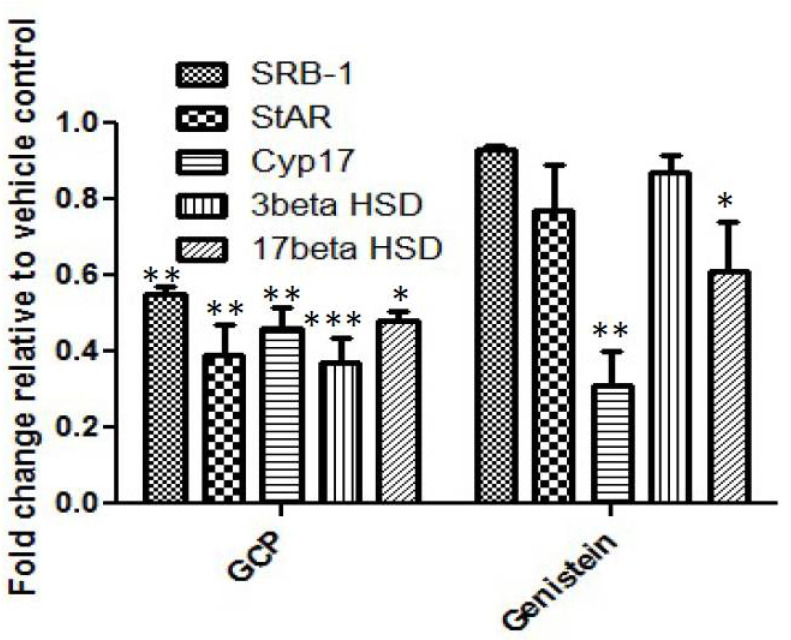
Quantitative real-time PCR analysis demonstrate that GCP and genistein can both downregulate CYP17 expression to a similar degree in LNCaP cells, and that GCP can also downregulate expression of other key molecules required for intracrine androgen synthesis. GCP mediated decreased expression of all the molecules required for intracrine androgen synthesis in LNCaP cells; GCP mediated a ~2.5-fold decrease in StAR (*p* < 0.001) and 3β HSD (*p* < 0.0001), and a ~2-fold decrease in SRB-1 (*p* < 0.001), Cyp17 (*p* < 0.001), 17β HSD (*p* < 0.01). Genistein decreased Cyp17 expression levels to a similar degree as GCP and fraction 40 (~3-fold decrease (*p* < 0.001)) and also mediated a decrease in 17β HSD expression (~1.7-fold decrease (*p* < 0.01)), however, genistein treatment did not cause a statistically significant difference in expression of SRB-1, StAR, or 3β HSD, the other molecules which are required for intracrine androgen synthesis to occur. (* = *p* < 0.01, ** = *p* < 0.001, *** = *p* < 0.0001).

Recent pre-clinical and clinical studies have demonstrated that some CaP cells can synthesize androgens (primarily testosterone and DHT) from steroid hormone precursors, e.g., cholesterol and cholesterol-3-SO_4_, and hence are not dependent upon testicular and/or adrenal androgens for the activation of AR and promotion of CaP cell proliferation and survival [27,28]. Importantly, it has been shown that intracrine androgen synthesis utilizing CYP17A can contribute to CaP disease progression [27]. To determine whether the anti-proliferative, pro-survival, and anti-AR effects of GCP are in part due to its ability to inhibit intracrine androgen synthesis, we first assessed the impact of GCP on steroid production by performing HPLC-MS analysis on conditioned media collected from LNCaP cells treated with GCP versus a vehicle control (Table 1). GCP treatment mediated a 3.3-fold decrease in testosterone levels, and reduced epitestosterone levels from 326 to 0. Alteration in the level of cholesterol, a precursor for intracrine androgen synthesis, was not observed, however, GCP mediated a 2.5-fold reduction in cholesterol-3-SO_4_, which is also a precursor for intracrine androgen synthesis. It is noteworthy that GCP treatment also affected levels of steroids involved in bile acid biosynthesis; GCP mediated a 2.5-fold increase in 7-keto-cholesterol, a steroid that can inhibit cholesterol 7-alpha-hydroxylase, the rate-limiting step in bile acid synthesis, an almost 14-fold decrease in taurodeoxycholicacid (TDCA), and reduced glycodeoxycholic acid levels from 102 to zero (Table 1). Subsequent quantitative real-time PCR (qPCR) analysis revealed that GCP treatment had a statistically significant impact on expression of all of the key molecules which are required for intracrine androgen synthesis in CaP cells (Figure 2); GCP mediated a ~2–3-fold decrease in the transcript levels of SRB-1, StAR, Cyp17, 3β HSD, 17β HSD. Treatment with genistein, the primary isoflavone present in GCP, caused a similar degree of inhibition of Cyp17 (~3-fold decrease) and a lesser decrease in expression levels of 17β HSD (~1.6-fold decrease), however, genistein treatment did not affect expression levels of SRB-1, StAR, or 3β HSD. The combined data demonstrate that GCP treatment can significantly reduce testosterone levels in CaP cells and indicate that this occurs via its ability to inhibit enzymes required for intracrine androgen synthesis.

### 3.3. GCP Fractionation and Cell Proliferation Assays show that GCP has Three Main Active Fractions

**Figure 3:**
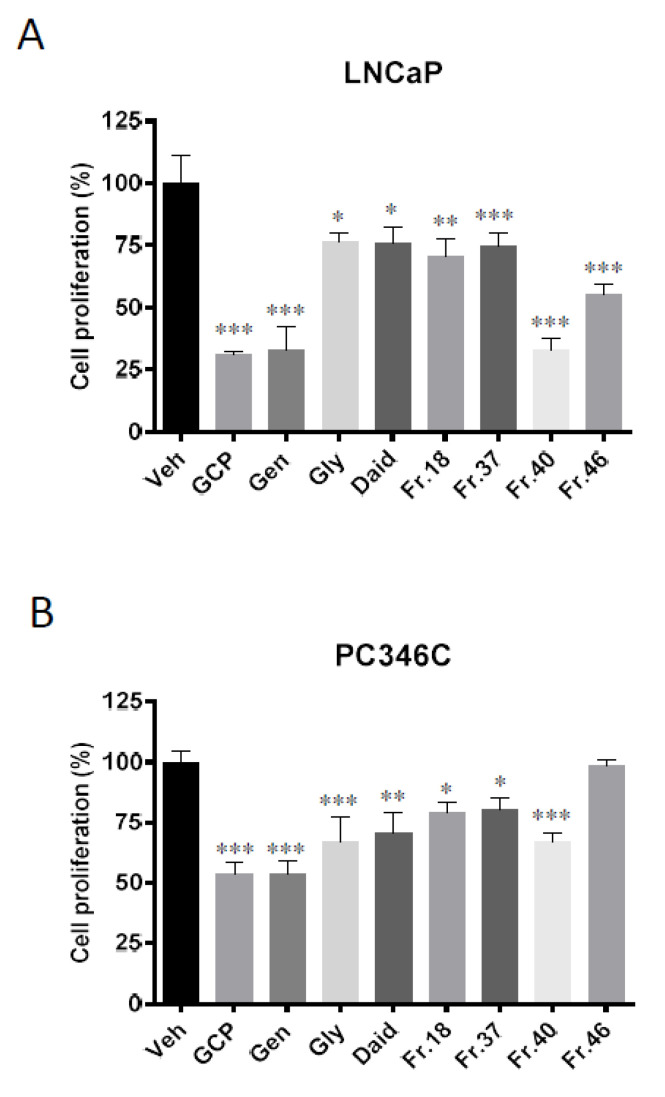
Fractionation of GCP followed by bioassay identifies fraction 40 as having the highest level of anti-proliferative activity in prostate cancer cell lines. Out of the 50 GCP fractions tested, only three fractions (fraction 18 (Fr.18), 37 (Fr.37), and 40 (Fr.40)) mediated a statistically significant decrease in the proliferation of both (**A**) LNCaP cells (~1.3-fold, ~1.3-fold, ~3.3-fold decrease, respectively) and (**B**) PC346C cells (~1.2-fold, ~1.2-fold, ~1.5-fold decrease, respectively) compared to the vehicle control (Veh). Fraction 46 (Fr.46) showed anti-proliferative activity in LNCaP cells (~2-fold decrease in proliferation) but not in PC346C cells. Genistein (Gen), daidzein (Daid), and glycitein (Gly), all of which are key components of GCP, also inhibited cell proliferation of both LNCaP (~3.3-fold, ~1.3-fold, ~1.3-fold, respectively) and PC346C cells (~1.9-fold, ~1.5-fold, ~1.5-fold, respectively). (GCP (100 µg/mL), genistein (9 µg/mL), daidzein (6 µg/mL), glycitein (2 µg/mL), GCP fractions (concentrations used were reflective of their concentration in 100 µg/mL GCP, as determined by LC-MS analysis)). (* = *p* < 0.01, ** = *p* < 0.001, *** = *p* < 0.0001).

Our group and others have previously demonstrated that GCP can cause the inhibition of CaP cell proliferation [9,10,13,14,15], however, the identity of the molecule(s) responsible for this activity is unknown. To identify which components of GCP are responsible for its biological activity, GCP was fractionated into 50 fractions using reverse-phase HPLC. All 50 fractions were dried, weighed and resuspended in ethanol: DMSO, 50:50 *v*/*v*, and then further diluted in PBS to achieve the required concentration for bioassay. Two CaP cell lines, LNCaP (derived from a lymph node metastasis of CaP) and PC-346C (derived from a patient with localized CaP), were used to identify which of the 50 fractions contributed to the ability of GCP to inhibit CaP cell proliferation. Proliferation assay data show that treatment of LNCaP cells with GCP, genistein, glycitein, daidzein, and fractions 18, 37, 40 and 46 (eluted at minutes 18, 37, 40 and 46, respectively) resulted in a statistically significant decrease in cell proliferation relative to the vehicle control (GCP, genistein, and fraction 40 all mediated a ~3.3-fold decrease, glycitein, daidzein, fraction 18, and fraction 37 mediated a ~1.3-fold decrease, and fraction 46 mediated a ~1.6-fold decrease (Figure 3A)). None of the other fractions affected LNCaP cell proliferation. The treatment of PC346C cells with GCP, genistein, glycitein, daidzein, and fractions 18, 37, and 40 resulted in a statistically significant decrease in proliferation relative to the vehicle control (GCP and genistein mediated a ~1.9-fold decrease, fraction 40 mediated a ~1.7-fold decrease, glycitein and daidzein mediated a ~1.5-fold decrease, and fractions 18 and 37 mediated a ~1.3-fold decrease (Figure 3B)). In contrast to LNCaP, treatment of PC346C cells with fraction 46, or with any of the other fractions, did not mediate a statistically significant decrease in cell proliferation. The combined data demonstrate that fractions 18, 37, and fraction 40 are the only fractions which have anti-proliferative activity in both LNCaP and PC346C cell lines. Two of these fractions, fractions 37 and 40, which had the highest activity and highest relative concentration of all fractions in GCP (HPLC analysis, supplementary data; Figure S1), were chosen for assessment by additional bioassay experiments and underwent NMR and LC-MS analysis.

### 3.4. HPLC, LC-MS, and NMR Analysis Show the Primary Constituent of Fraction 40 is Genistein, and that the Primary Constituents of Fraction 37 is Daidzein

HPLC analyses demonstrated that fraction 40 has the same retention time as genistein and that fraction 37 has same retention time as daidzein (supplementary data; Figure S1), indicating that these are the primary constituents of these fractions. Crude GCP run on UPLC-MS (Figure 1) confirmed the presence of genistein, daidzein, glycitein in these fractions and showed the presence of other unknown compounds. The composition of fractions 37 and 40 was further elucidated by 1D ^1^H NMR and 2D ^1^H-^13^C HSQC NMR analyses (Supplemental Data; Figures S2–S7). Published data, retention time, and photodiode array detector were taken into account as part of the analyses. Comparison of fraction 37 and fraction 40 with their respective standards (genistein, daidzein, and/or glycitein) demonstrated that the same R-Bz-OH moieties were present, and that genistein and daidzein are present in fractions 37 and 40 but with differing ratios; fraction 40 is primarily composed of genistein with trace amounts of daidzein, and fraction 37 is primarily composed of daidzein with trace amounts of genistein. Fraction 40 also contains trace amounts of glycitein. Our analyses revealed that other aliphatic compounds are present at low levels in both fractions, and that higher levels of these are present in fraction 37 compared to fraction 40.

**Figure 4:**
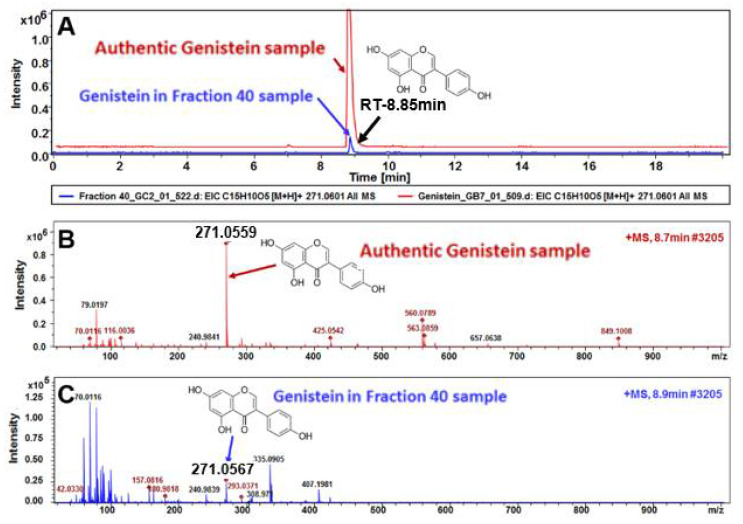
LC-MS validation of genistein as the major component of fraction 40 (**A**): Extraction ion chromatogram (EIC) of authentic genistein sample compared with genistein EIC from fraction 40 reveals a perfect retention time (RT) match at around 8.85 min, as expected, confirming the correct identification of the compound. (**B**,**C**): The compound spectra of authentic genistein (**B**) and genistein in fraction 40 (**C**) are depicted. The [M + H] = 271.055 ± 0.001 *m/z* of the identified compound is the same with that of the authentic genistein. The data profile analysis (**A**–**C**) depicts a representation of six replicates with high confidence of reproducibility.

To validate that genistein is the major compound of fraction 40, we ran an authentic genistein with fraction 40 using an advanced LC-MS data analysis (Figure 4). The retention time (RT) of extracted ion chromatogram (EIC) of genistein compound in the authentic as well as fraction 40 base peak chromatograms (BPC) are perfectly overlapped, confirming the perfect identification of this compound in fraction 40. Moreover, the analysis of compound spectral profile of this EIC molecule in the authentic as well as fraction 40 samples confirm the molecular mass of the compound as well as the expected MS spectra of the compound in fraction 40 compared to the authentic sample. This advanced high-resolution LC-QTOF MS data analysis validates genistein as being the primary constituent of fraction 40.

### 3.5. Genistein is Responsible for the Majority of GCP’s Anti-Proliferative, Anti-Androgen Receptor, and Pro-Apoptotic Activity

**Figure 5:**
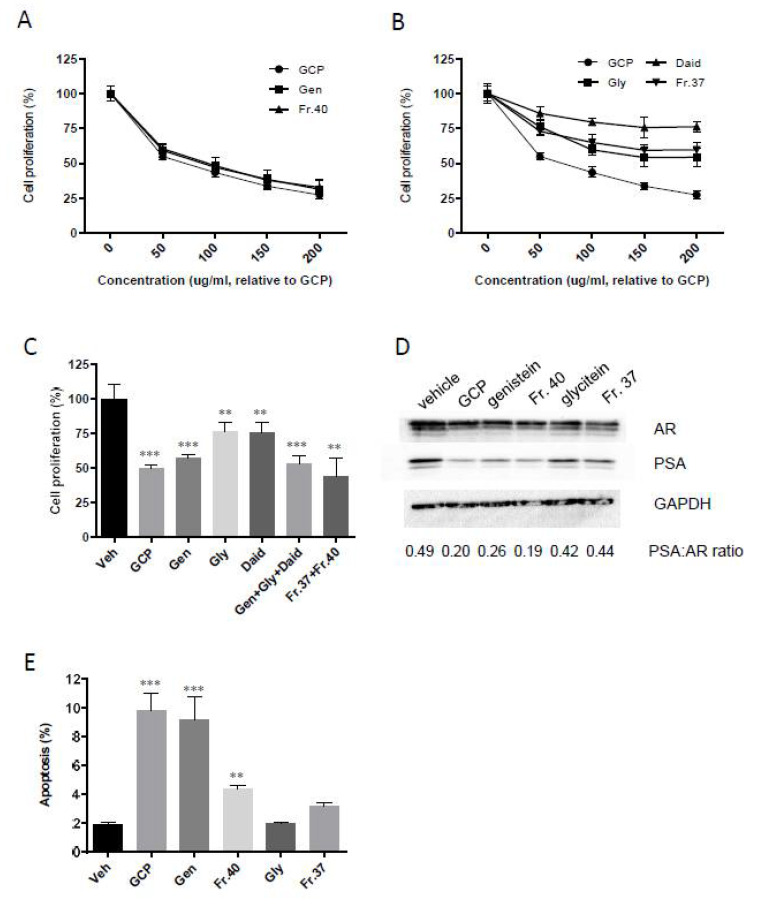
GCP and genistein are equally effective in terms of their ability to inhibit prostate cancer cell proliferation, decrease androgen receptor expression, decrease PSA levels, and promote cell apoptosis. All of the biomolecules (GCP, genistein, daidzein, glycitein) and fractions (fractions 37 and 40) being tested caused a dose-dependent reduction is LNCaP cell proliferation (**A**,**B**). Our data further confirm that GCP, genistein, and fraction 40 have similar levels of anti-proliferative activity (**A**). Combining genistein, glycitein, and daidzein, or combining fractions 37 and 40, did not further decrease cell proliferation compared to treatment with GCP or genistein as single agents (**C**). GCP, genistein, and fraction 40 decreased PSA activity to a similar degree (~2.5-fold decrease (**D**). GCP, genistein, and fraction 40 also caused a significant increase in apoptosis (**E**), however, while GCP and genistein caused a ~5-fold increase in apoptosis, fraction 40 mediated only a ~2-fold increase. Note that, for these bioassay experiments, the concentration of the GCP fractions, genistein, glycitein was adjusted to reflect their relative concentration in GCP (100 ug/mL), hence the *x*-axis is labelled ‘concentration (ug/mL relative to GCP)’. (**C**–**E**); GCP (100 µg/mL), genistein (9 µg/mL), daidzein (6 µg/mL), glycitein (2 µg/mL), GCP fractions (concentrations used were reflective of their concentration in 100 µg/mL GCP as determined by LC-MS analysis)). ** = *p* < 0.001, *** = *p* < 0.0001).

Both fraction 40 and fraction 37 inhibited the proliferation of LNCaP cells in a dose-dependent manner (Figure 5A,B). Fraction 37 caused a lesser inhibition compared to GCP (Fraction 37 IC50 > 200 ug/mL, GCP IC50 ~75 ug/mL), while fraction 40 decreased cell proliferation to a similar extent to GCP and genistein (IC50 for all three molecules is ~75 ug/mL). These data, combined with our UPLC-MS and NMR data, indicate that genistein is primarily responsible for the anti-proliferative activity of fraction 40 and GCP.

To help determine whether the effects of each isoflavone were additive, combination experiments were performed (Figure 5C). The combination of genistein, glycitein, and daidzein as well as the combination of fraction 37 and 40 inhibited cell proliferation to the same extent as GCP and genistein as single agents (~2-fold reduction in cell proliferation for all of these treatments). These data indicate that the effects of genistein, daidzein, and glycitein are not additive, although more extensive analyses involving isobologram and combination index studies are needed to prove this.

Previous studies by our group and others have demonstrated that GCP can decrease AR levels and reduce AR activity [14,15,29,30]. In the current study, immunoblot analysis of AR and PSA was performed to determine which component of GCP is responsible for this activity (Figure 5D). Our data show that GCP, fraction 40, and genistein mediate a similar reduction in AR expression levels (~1.6-fold decrease for all three treatments) as well as a similar reduction in PSA levels (4.1-fold, 3.0-fold, and 4.2-fold decrease, respectively (PSA expression levels are an indicator of AR activity)). Decreases were also observed in the PSA:AR ratios for these treatments (2.5, 2.0, and 2.5-fold decrease compared to vehicle control, respectively) (full blots and densitometry data can be found in Supplementary Figure S8). These data demonstrate that fraction 40 and genistein are equally effective as GCP in mediating inhibition of AR activity. Treatment with glycitein and fraction 37 also mediated decreased AR and PSA levels, but to a lesser extent (glycitein mediated a 1.5-fold decrease in AR, a 1.7-fold decrease in PSA, and a 1.2-fold decrease in PSA:AR ratio compared to vehicle control, while fraction 37 mediated a 1.9-fold decrease in AR, a 2.1-fold decrease in PSA, and 1.1-fold decrease in PSA:AR ratio). Flow cytometry analyses (Figure 5E) demonstrated that GCP and genistein both mediate a ~5-fold increase in apoptosis. In contrast, fraction 40 mediated only a ~2-fold increase. These data suggest that there may be constituents present in fraction 40 which counter the pro-apoptotic effects of genistein. Glycitein and fraction 37 did not mediate a statistically significant increase in apoptosis.

## 4. Discussion

Our data demonstrate that GCP can reduce testosterone levels through inhibiting intracrine androgen synthesis in CaP cells. We also identify genistein as being the primary constituent of GCP that contributes to its ability to inhibit CYP17 and to inhibit CaP cell proliferation and promote apoptosis. To our knowledge, the ability of GCP and genistein to inhibit intracrine androgen synthesis has not previously been reported.

**Figure 6:**
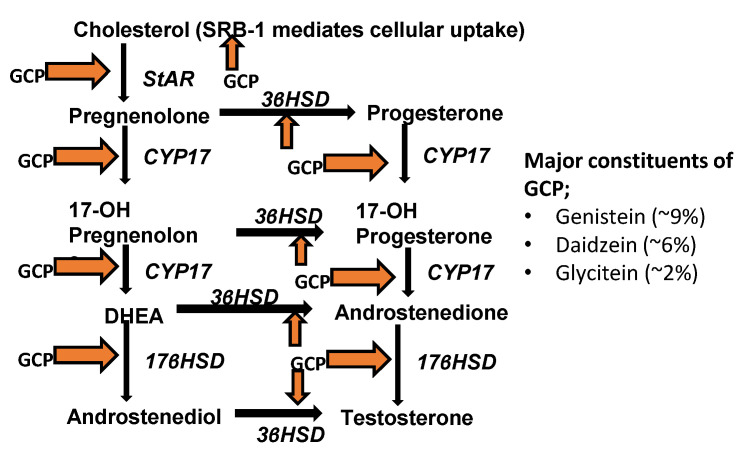
Overview of testosterone synthesis. Our data indicate that GCP is able to inhibit the expression of several enzymes required for testosterone synthesis, including SRB-1, StAR, Cyp17, 3β HSD, and 17β HSD. The primary constituents of GCP are genistein (~9%), daidzein (~6%), and glycitein (~2%).

Intracrine androgen synthesis has been shown to occur in many CaP patients and can allow for continued activation of AR after initiation of ADT, and thereby contribute to ADT failure [27]. There are multiple steps required for intracrine androgen synthesis; SRB-1, StAR, Cyp17, 3β HSD, and 17β HSD all play a key role; SRB-1 is necessary for cholesterol uptake, StAR transports cholesterol into the inner mitochondrial membrane, and 3β-HSD, CYP17 and 17βHSD1 are enzymes required for androgen synthesis (Figure 6). It is noteworthy that the inhibition of CYP17 (which was inhibited to a similar degree by GCP and genistein) has been shown to cause a significant decrease in androgen synthesis by CaP cells, and that CYP17 is the target of Abiraterone, a drug that was recently approved to treat CaP patients [31]. It is very likely that the ability of GCP to inhibit intracrine androgen synthesis at least in part accounts for the reduction in AR activity that our group and others have observed following treatment of CaP cells with GCP [9,10,11,12,13,14,15]. Further studies will be necessary to determine whether GCP can inhibit intracrine androgen synthesis in an in vivo setting. If it can, then GCP, like Abiraterone, may have clinical utility in some CaP patients. It is noteworthy that GCP has a favorable toxicity profile in CaP patients [11].

Our combined data identify genistein as the primary constituent of GCP responsible for its biological activity, including its ability to inhibit AR expression and activity and to inhibit CYP17. Several studies have shown that genistein can inhibit CaP cell proliferation and several mechanisms have been proposed [32,33]. Ajdzanovic et al. demonstrated that genistein can inhibit AR activity by decreasing membrane fluidity and causing immobilization of lipid rafts containing AR [30], and Vickman et al. demonstrated that genistein can inhibit AR activity by inhibiting Hsp90, an AR chaperone [29]. Our data indicate that inhibition of intracrine androgen synthesis also contributes to its anti-AR activity. While our study indicates that GCP and genistein have similar levels of biological activity in CaP cell lines and they both inhibit CYP17 to a similar degree, a major advantage of using GCP instead of genistein in patients is that GCP has far better bioavailability; during the manufacture of GCP, its constituent isoflavones are metabolized into the aglycone form which can more easily cross the GI tract compared to the naturally occurring glycone form [19,34,35]. Achieving high levels of genistein in patients is important because it is known that there is a dose-dependent relationship between serum concentrations of genistein and reduced CaP risk (the Japanese cohort study; 14,105 men followed for 9–11 y [36]). Certainly, another strategy to consider here is deliver of genistein via nanoparticles as described by Song et al. and others [37,38,39]. In addition to improving absorption, the use of nanoparticles to deliver pharmacologic agents can also allow for controlled release and targeted delivery [40].

We demonstrate that GCP and genistein cause a decrease in AR expression levels as well as AR activity. In previous studies, we demonstrated this was not due to de-repression of AR via inhibition of the PI3K-Akt-mTOR pathway and the mechanism responsible remains unclear [14]. Interestingly, other Cyp17 inhibitors, including Abiraterone, have been shown to decrease AR expression levels suggesting that it is possible that Cyp17 inhibition may play a role [41,42]. Our current study confirmed our previous finding that GCP and genistein can cause apoptosis [14]. Our prior studies and studies by others have determined that GCP and genistein can mediate apoptosis via AR-independent mechanisms. For example, Shafiee et al. have demonstrated that genistein can promote apoptosis in PC3 cells, a cell line which lacks AR [43], and our group demonstrated that GCP mediates apoptosis via inhibition of mTOR14. Grossebrummel et al. found that genistein can mediate increased apoptosis of cancer cells via increasing expression levels of caspase 3, 7, and 12, as well as by causing increased cellular stress [44]. Lastly, data from a genome-wide DNA methylation and gene expression study identified multiple non-AR-related genes as being impacted by genistein, including *NOTCH3*, *JAG1*, *ADCY4* and *NEU1* [45].

In addition to showing that GCP mediates decreased testosterone levels, LC-MS analysis identified GCP-mediated changes in the levels of other steroids. For example, GCP caused a 2.5-fold reduction in cholesterol sulfate (CS), a molecule that has been identified as a potential CaP progression marker and a precursor for testosterone synthesis via the intracrine androgen synthesis pathway [28]. Interestingly, clinical studies have shown that CS is observed almost exclusively in cancerous tissues [46]. We also found GCP has a dramatic effect on steroids associated with bile acid metabolism. Treatment with GCP resulted in 2.5-fold increase in 7-keto-cholesterol, a steroid that can inhibit cholesterol 7-alpha-hydroxylase, the rate-limiting step in bile acid biosynthesis, and an almost 14-fold decrease in taurodeoxycholicacid (TDCA) levels, a cytotoxic bile acid that is known to promote cell proliferation [47,48]. Bile acids have also been reported to induce apoptosis via the disruption of mitochondrial function, ligand-independent activation of death receptor pathways, and by modulation of members of the Bcl2 protein family [49,50]. Further studies will be needed to confirm these observations and to determine whether these steroids play a role in prostate carcinogenesis.

Our combined data support further investigation of the ability of GCP to inhibit intracrine androgen synthesis. A first step will be to conduct mechanistic studies to fully elucidate the mechanism(s) by which GCP and genistein inhibit intracrine androgen synthesis and impact CaP cell proliferation and apoptosis. We also plan to conduct in vivo studies in CaP animal models. A long-term goal will be to determine whether GCP can be used to reduce ADT failure rates in CaP patients and thereby improve survival rates. Our identification of the active component of GCP as well as GCP’s favorable toxicity profile will improve the feasibility of implementing such clinical studies.

## Figures and Tables

**Table 1:** Steroid composition analysis demonstrates that GCP can downregulate testosterone synthesis as well as the synthesis of other steroids by LNCaP cells. Steroid composition analysis demonstrates GCP can downregulate testosterone synthesis as well as the synthesis of other steroids by LNCaP cells. Data are shown as means (SD). The experiment was performed using triplicate samples.

Metabolites	Vehicle (Pg/30 Million Cells (SD))	GCP (Pg/30 Million Cells (SD))	*p*-Value
**Testosterone**	46 (4.1)	14 (1.2)	*p* < 0.001
**Epitestosterone**	326 (7.2)	0 (0)	*p* < 0.001
**Cholesterol**	440,339 (50.3)	454,832 (90.9)	*p* < 0.001
**Cholesterol-3-SO4**	431,845 (73.1)	209,803 (29.9)	*p* < 0.001
**7-Keto-Cholesterol**	126,973 (67.6)	322,883 (87.9)	*p* < 0.001
**Taurodeoxycholic acid**	326 (15.8)	23 (1.5)	*p* < 0.001
**Glycodeoxycholic acid**	102 (5.9)	0 (0)	*p* < 0.001

## Supplementary Materials

The following are available online at https://www.mdpi.com/2227-9059/8/8/282/s1, Figure S1: Reverse Phase HPLC analysis of GCP, Figure S2: 1D ^1^H NMR analysis, Figure S3: 2D ^1^H-^13^C HSQC NMR analysis of fraction 37, Figure S4: 2D ^1^H-^13^C HSQC NMR analysis of fraction 40, Figure S5: 2D ^1^H-^13^C HSQC NMR analysis of genistein, Figure S6: 2D ^1^H-^13^C HSQC NMR analysis of glycitein, Figure S7: 2D ^1^H-^13^C HSQC NMR analysis of daidzein, Figure S8: Western blot quantification

## Abbreviations

The abbreviations used are:

ADT	Androgen deprivation therapy
AR	Androgen receptor
CAM	Complimentary alternative medicine
CaP	Prostate cancer
CRPC	Castration-resistant prostate cancer
CS	Cholesterol sulfate
GCP	Genistein combined polysaccharide
HPLC	High-Performance liquid chromatography
HSQC NMR	Heteronuclear multiple bond correlation nuclear magnetic resonance
NMR	Nuclear magnetic resonance
qPCR	Quantitative real-time polymerase chain reaction
TDCA	Taurodeoxycholicacid
UPLC-MS	Ultra-Performance liquid chromatography-mass spectrometry
