# Supplementary material for: Genistein Combined Polysaccharide (GCP) Can Inhibit Intracrine Androgen Synthesis in Prostate Cancer Cells
Source: Biomedicines. 2020 Aug 11;8(8):282. doi: 10.3390/biomedicines8080282 (PMC7460199; doi:10.3390/biomedicines8080282)
Supplement: Supplementary file 1 [file biomedicines-08-00282-s001.pdf]

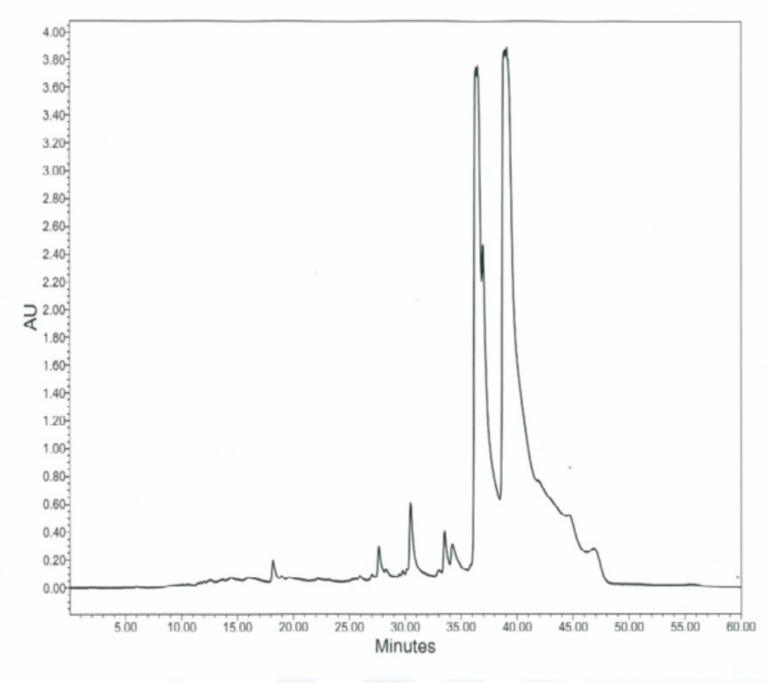

S1: reverse phase HPLC analysis of GCP

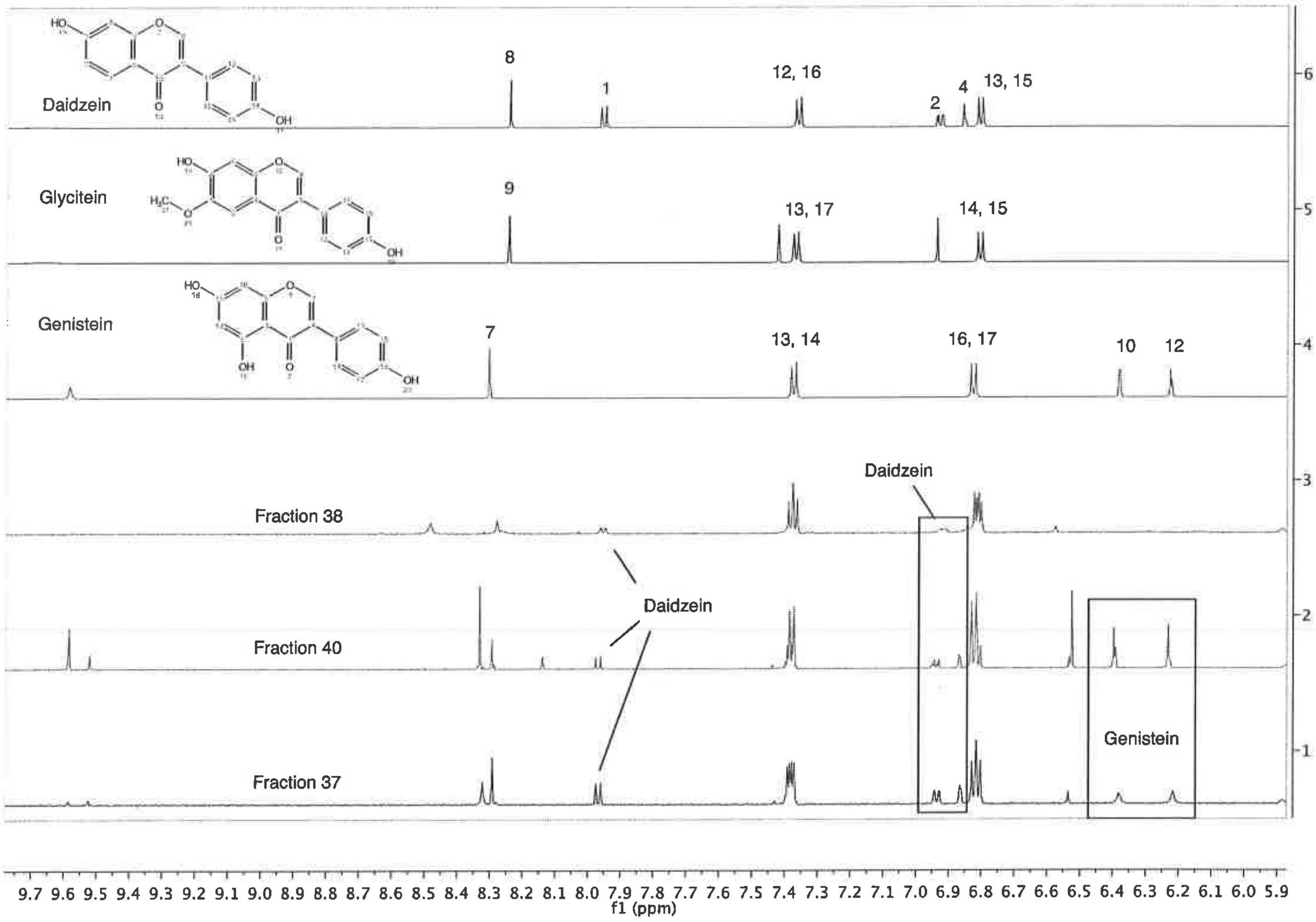

S2: 1D 1H NMR Analysis

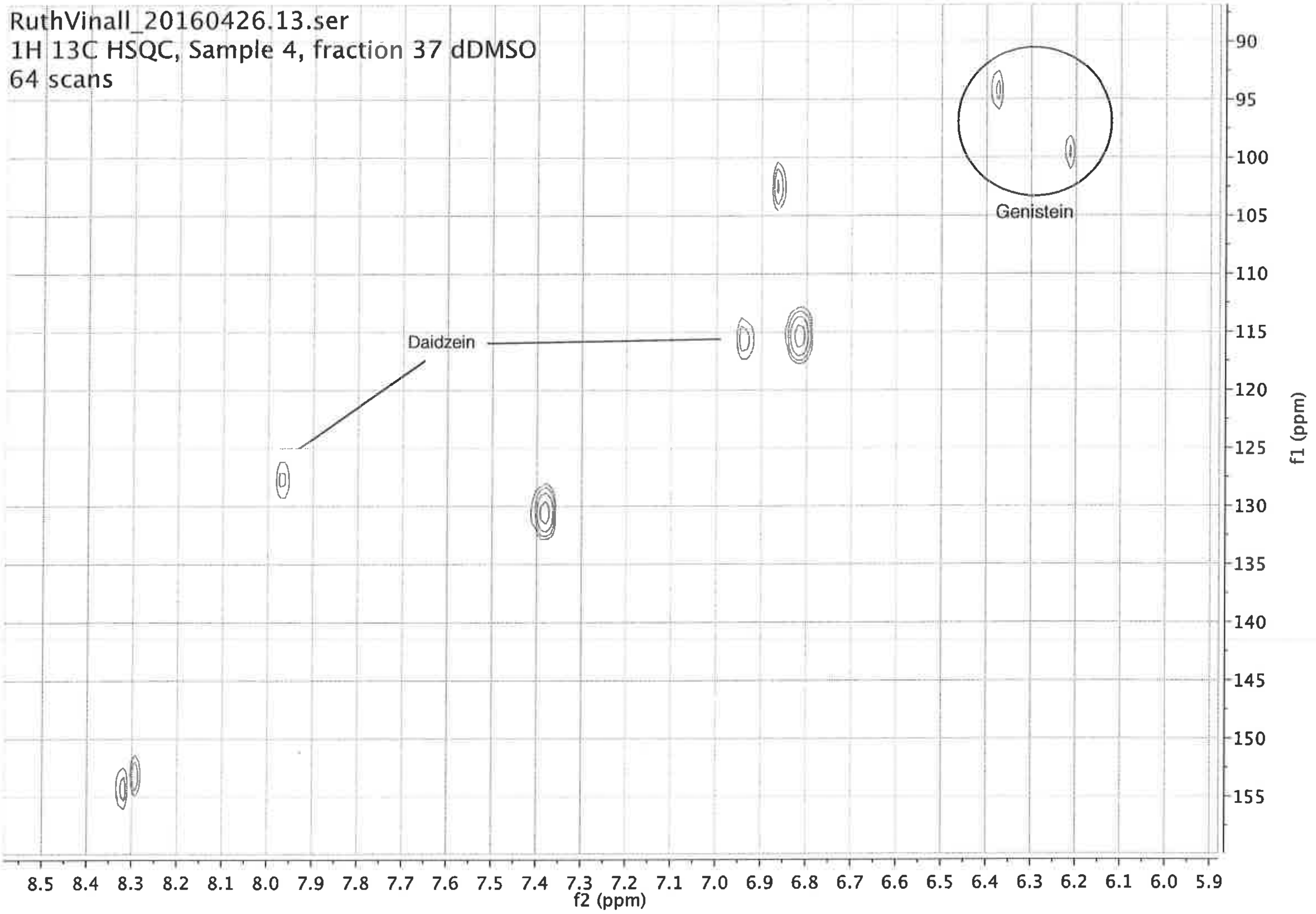

S3: 2D 1H-13C HSQC NMR analyses, fraction 37

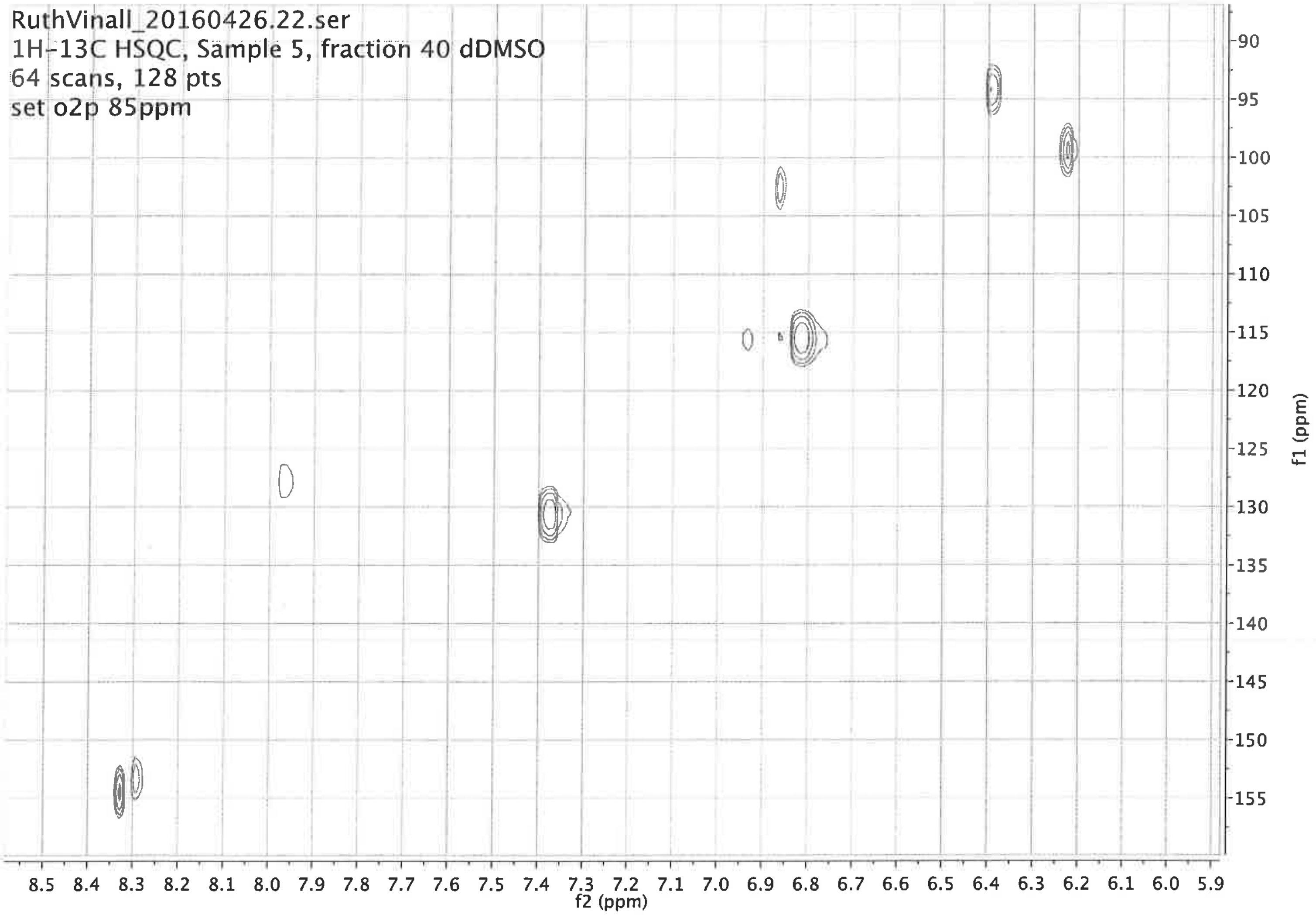

S4: 2D 1H-13C HSQC NMR analyses, fraction 40

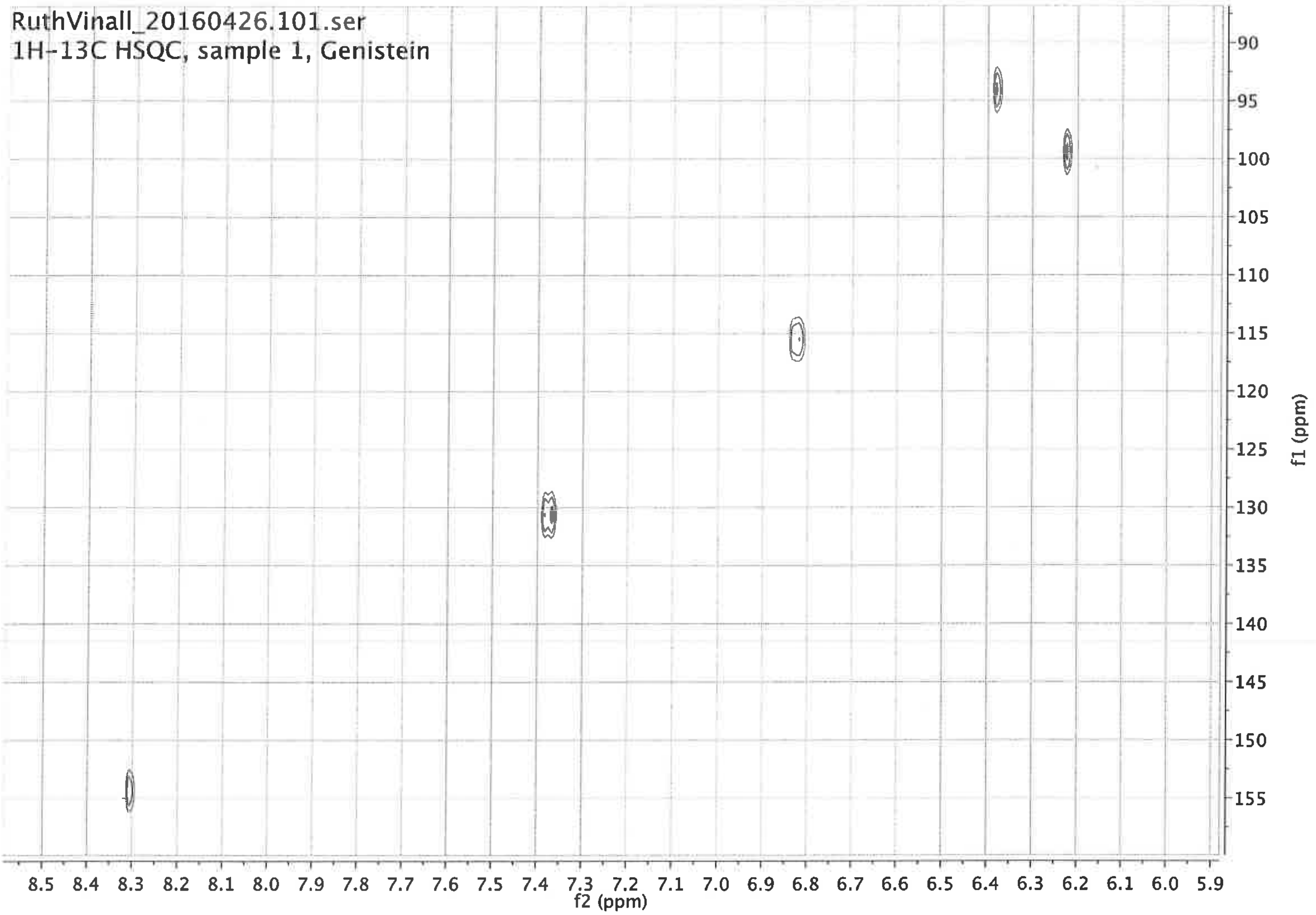

S5: 2D 1H-13C HSQC NMR analyses, Genistein

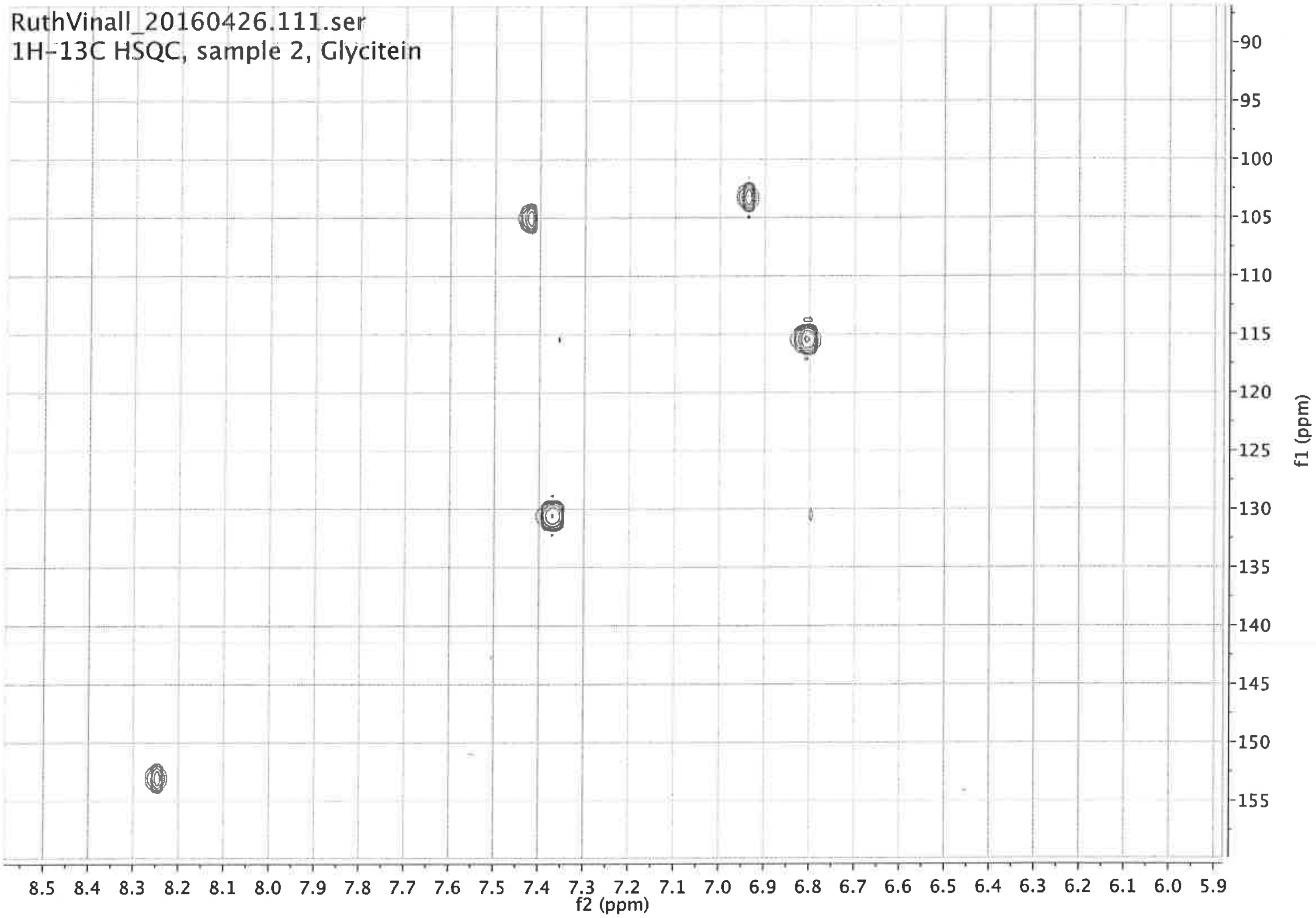

S6: 2D 1H-13C HSQC NMR analyses, glycitein

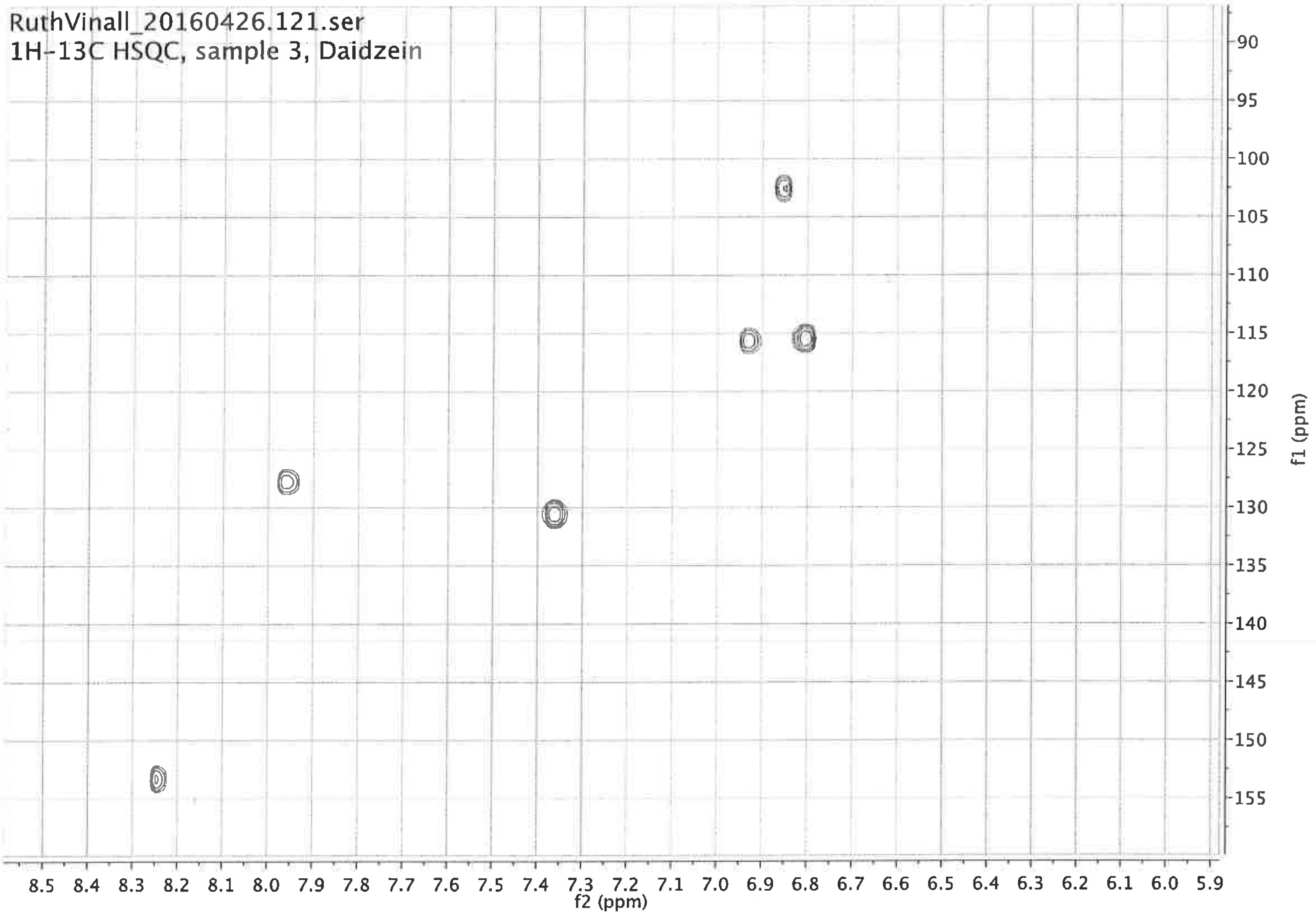

S7: 2D 1H-13C HSQC NMR analyses, daidzein

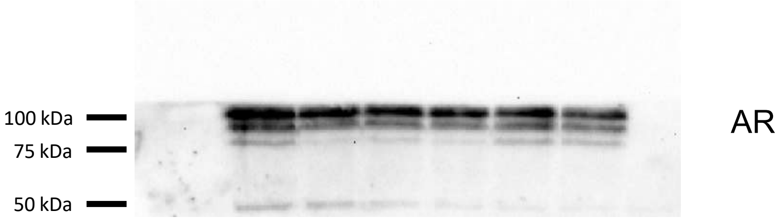

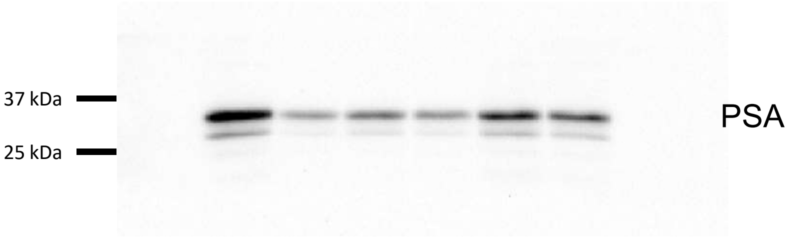

	V	GCP	Gen	Fr.40	Gly	Fr.37
AR, densitometry	17691.69	10928.16	11291.06	10687.87	12138.36	9131.21
PSA, densitometry	8720.07	2141.49	2936.74	2063.08	5215.76	4088.46
PSA:AR ratio	0.49	0.20	0.26	0.19	0.42	0.44

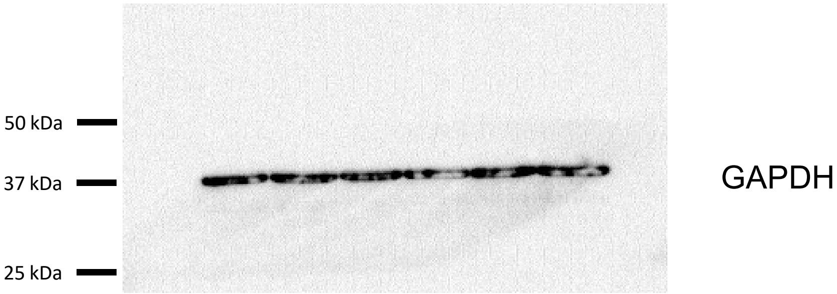

	V	GCP	Gen	Fr.40	Gly	Fr.37
GAPDH, densitometry	12068.16	12498.19	12153.94	10654.65	12010.76	11765.89

S8: Western blot quantification
